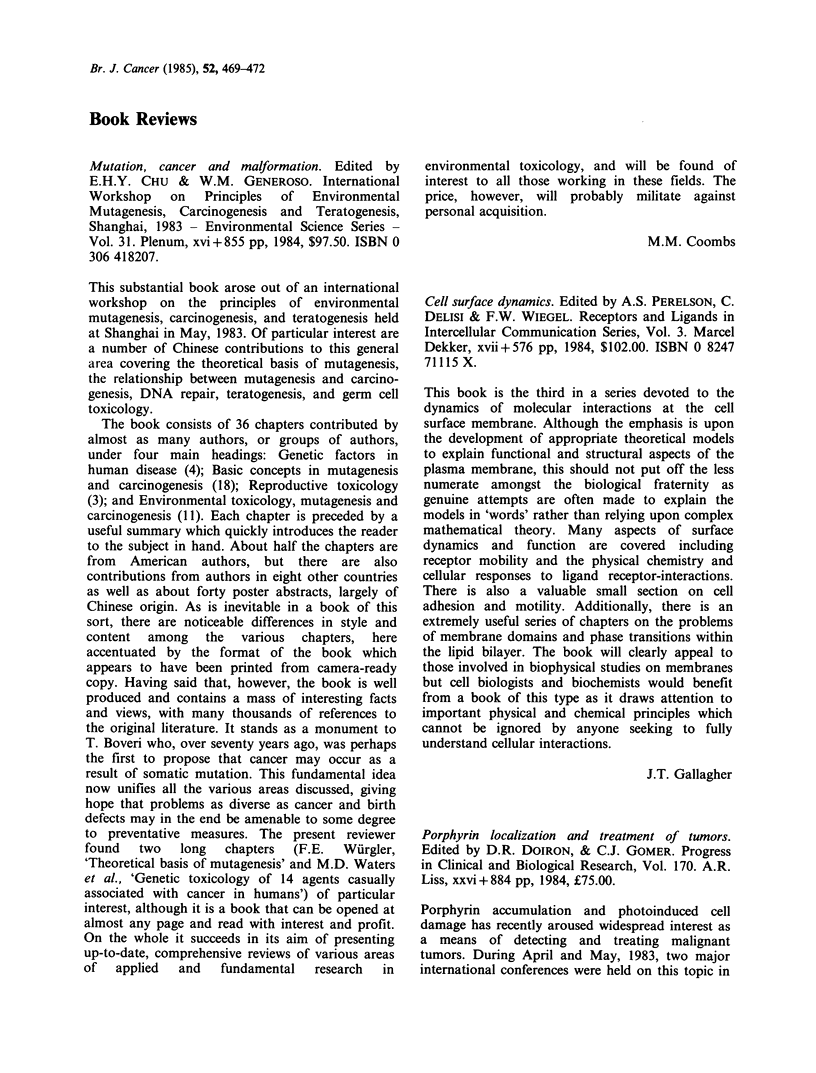# Cell surface dynamics

**Published:** 1985-09

**Authors:** J.T. Gallagher


*Cell surface dynamics*. Edited by A.S. PERELSON, C. DELISI & F.W. WIEGEL. Receptors and Ligands in Intercellular Communication Series, Vol. 3. Marcel Dekker, xvii+576 pp, 1984, $102.00. ISBN 0 8247 71115 X.

This book is the third in a series devoted to the dynamics of molecular interactions at the cell surface membrane. Although the emphasis is upon the development of appropriate theoretical models to explain functional and structural aspects of the plasma membrane, this should not put off the less numerate amongst the biological fraternity as genuine attempts are often made to explain the models in 'words' rather than relying upon complex mathematical theory. Many aspects of surface dynamics and function are covered including receptor mobility and the physical chemistry and cellular responses to ligand receptor-interactions. There is also a valuable small section on cell adhesion and motility. Additionally, there is an extremely useful series of chapters on the problems of membrane domains and phase transitions within the lipid bilayer. The book will clearly appeal to those involved in biophysical studies on membranes but cell biologists and biochemists would benefit from a book of this type as it draws attention to important physical and chemical principles which cannot be ignored by anyone seeking to fully understand cellular interactions.

J.T. Gallagher